# Reference-Free Microsatellite Instability Detection from Tumor Sequencing Using Intrasample Variability Modeling

**DOI:** 10.34133/csbj.0219

**Published:** 2026-09-09

**Authors:** Georgios Vlachos, Tina Moser, Mitesh Patel, James R. White, Carina Pischler, Lisa Glawitsch, Thomas Bauernhofer, Philipp Jost, Leo Edlinger, Karl Kashofer, Jochen B. Geigl, Luis A. Diaz, Ellen Heitzer

**Affiliations:** ^1^ Institute of Human Genetics, Diagnostic & Research Center for Molecular BioMedicine, Medical University of Graz, Austria.; ^2^ Division of Solid Tumor Oncology, Memorial Sloan Kettering Cancer Center, New York, NY, USA.; ^3^ Resphera Biosciences, Baltimore, MD, USA.; ^4^ Division of Oncology, Department of Internal Medicine, Medical University of Graz, Graz, Austria.; ^5^ Diagnostic and Research Institute of Pathology, Medical University Graz, Graz, Austria.

## Abstract

Microsatellite instability (MSI) is a predictive biomarker in several tumor types. However, many next-generation sequencing-based callers require matched normal samples, reference panels, or pretrained models, limiting their portability across assays and sequencing centers. We developed PROMIS (PROfiling of Microsatellite InStability), a tumor-only, reference-free pipeline that uses a discrete mixture model to characterize intrasample repeat-length distributions at predefined microsatellite loci. Locus-level classifications are then aggregated into a continuous MSI score. We benchmarked PROMIS in colorectal (CRC), endometrial (UCEC), and gastric (STAD) cancers from The Cancer Genome Atlas. PROMIS achieved an overall area under the receiver operating characteristic curve (AUC) of 0.995 and cohort-specific AUCs of 1.00 in CRC and stomach adenocarcinoma and 0.999 in uterine corpus endometrial carcinoma, comparable to established tools despite not using matched normals or pretrained models. Subsampling demonstrated robust performance with substantially fewer loci. In silico dilution showed progressively reduced MSI–microsatellite-stable discrimination, with the pooled AUC declining from 0.83 at 10% tumor fraction to 0.53 at 1%. At low tumor fractions, tumor-type-specific baseline microsatellite variability increasingly influenced PROMIS scores. Finally, in prostate and CRC cell-free DNA cohorts, including Illumina TSO500 data and an 18-gene panel, PROMIS yielded MSI scores concordant with orthogonal tissue- and panel-based classifications across the evaluated Illumina-based sequencing contexts. Accordingly, the present validation should be considered limited to Illumina-based sequencing platforms. PROMIS is intended to complement existing genomic profiling workflows by enabling MSI assessment from sequencing data already generated for broader molecular analyses. Prospective clinical validation remains necessary before clinical implementation.

## Introduction

Microsatellite instability (MSI) is a key genomic biomarker arising from defects in the DNA mismatch repair (MMR) system and is widely used in molecular diagnostics, prognosis, and therapeutic decision-making. MSI refers to insertion/deletion mutations occurring within short tandem repeat regions throughout the genome, which arise when MMR deficiency (dMMR) fails to correct replication-associated errors. Beyond microsatellite alterations, dMMR generates a hypermutated phenotype characterized by increased neoantigen burden and pronounced immune infiltration. This biological context underlies the strong clinical responses of MSI-high (MSI-H) or dMMR tumors to immune checkpoint inhibitors (ICIs), establishing MSI as a key predictive biomarker in modern oncology [1,2]. MSI-H occurs across multiple tumor types, most frequently in colorectal cancers (CRCs)—often associated with Lynch syndrome—as well as endometrial and gastric cancers, and at lower frequencies in a broad range of other solid malignancies [3].

Traditionally, MSI detection has relied on polymerase chain reaction fragment analysis of microsatellite loci and immunohistochemistry for MMR protein expression [4]; however, the increasing adoption of next-generation sequencing has created opportunities for computational approaches that infer MSI directly from sequencing data. Despite substantial progress, existing MSI-calling methods remain limited by factors such as sequencing depth requirements, platform dependence, reduced robustness in heterogeneous tumor samples, and, in some cases, the need for corresponding normal tissue.

Early MSI-calling algorithms such as MSIsensor and MANTIS achieved high accuracy by comparing microsatellite repeat-length distributions between tumor and matched-normal samples [5,6], but their dependence on normal controls limits clinical applicability. Tumor-only approaches, including MSIsensor-pro [7], partially addressed this constraint using panels of normals or statistical modeling of polymerase slippage. Machine learning methods such as MiMSI [8] improved sensitivity in challenging samples but rely on pretrained models that may not generalize across sequencing platforms or populations. Additionally, many current tools reduce MSI to a binary classification, limiting biological interpretability. Specialized tools like MSICare achieve excellent accuracy in specific cancer types but are not broadly applicable [9]. Consequently, a reference-free, tumor-only, and interpretable approach remains lacking.

To address this gap, we developed PROMIS (PROfiling of Microsatellite InStability), a reference-free MSI-calling pipeline that detects instability directly from sequencing data from tissue or plasma without matched normals, external reference cohorts, or model training.

## Results

### Overview of the PROMIS pipeline

**Fig. 1.**
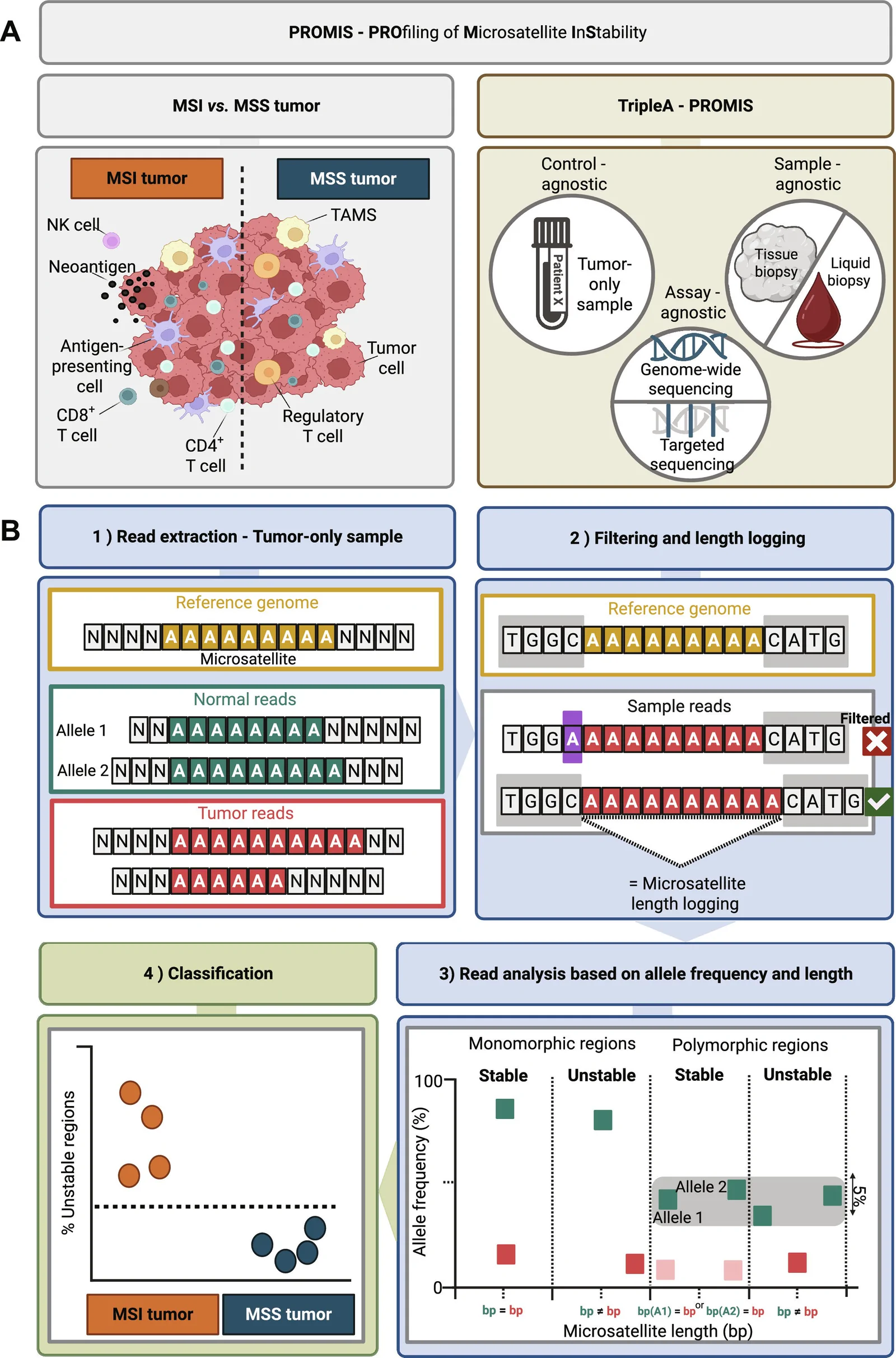
Overview of the PROMIS pipeline for tumor-only microsatellite instability (MSI) detection from next-generation sequencing data. PROMIS (PROfiling of Microsatellite InStability) is a control-agnostic and sample-agnostic framework with assay-adaptable locus selection applicable to both tissue and liquid biopsy samples and compatible with genome-wide (whole-exome sequencing and whole-genome sequencing) or targeted sequencing panels. (A) Left: Schematic representation of MSI *vs.* microsatellite-stable (MSS) tumors, highlighting the immunogenic neoantigen landscape and tumor microenvironment. Right: The design principles support application across sample types and the evaluated Illumina-based assays; Ion Torrent applicability was not demonstrated. (B) PROMIS workflow steps. (1) Reads overlapping predefined microsatellite loci are extracted from tumor-only samples. (2) Reads undergo stringent quality filtering, where an “X” indicates a filtered-out read and a “✓” a retained read, followed by microsatellite length logging. (3) Allele frequency and length distributions are analyzed. The x-axis represents microsatellite length in base pairs (bp): bp = bp denotes equal lengths across alleles (stable), while bp ≠ bp denotes unequal lengths indicative of instability. Both monomorphic regions (normally uniform repeats) and polymorphic regions (with natural allelic variation) are modeled. A discrete mixture modeling approach distinguishes stable loci, exhibiting expected polymorphisms, from unstable loci with multiple distinct allele populations. (4) The proportion of unstable loci is calculated, enabling robust binary classification into MSS and MSI-high tumors. NK, natural killer; TAMS, tumor-associated macrophages.

PROMIS is implemented as an automated and reproducible Snakemake workflow compatible with whole-genome sequencing (WGS), whole-exome sequencing (WES), and targeted sequencing panels [10]. PROMIS treats each tumor as its own internal control by modeling repeat-length distributions at predefined microsatellite loci using a discrete mixture modeling framework [11] (Fig. 1A). This approach distinguishes stable from unstable loci while accounting for natural polymorphism and sequencing noise, enabling transparent locus-level classification alongside a global MSI score. Its modular architecture supports both tissue and circulating cell-free DNA (cfDNA) analyses through integrated read-level filtering, locus extraction, mixture-model fitting, and sample-level scoring.

Binary sequence Alignment Map (BAM) or Compressed Reference-oriented Alignment Map (CRAM) files are processed by extracting reads overlapping predefined microsatellite loci, removing low-confidence reads and alignment artifacts, modeling repeat-length distributions at each locus using a discrete mixture model to identify multimodal allele populations indicative of instability, and aggregating locus-level classifications into a global MSI score representing the proportion of unstable loci within each sample (Fig. 1B).

Benchmarking was performed using 702 high-quality exonic microsatellite loci curated from the MOSAIC reference dataset, ensuring consistent locus representation across sequencing assays [12]. Alternatively, a preprocessing module automatically identifies confidently covered loci for the sequencing design and reference genome used, enabling straightforward application to custom capture panels. The workflow additionally reports read-level quality metrics, locus-level instability calls, and allele-length distribution plots. WES datasets are processed in <2 min, with runtime scaling approximately linearly with the number of loci analyzed, enabling efficient high-throughput analyses.

### Performance of PROMIS across tumor types

Performance was benchmarked using The Cancer Genome Atlas (TCGA) CRC, endometrial (uterine corpus endometrial carcinoma [UCEC]), and gastric (stomach adenocarcinoma [STAD]) cancer cohorts. A consensus classification of MSIsensor and MANTIS served as the reference standard, with discordant cases excluded.

**Fig. 2.**
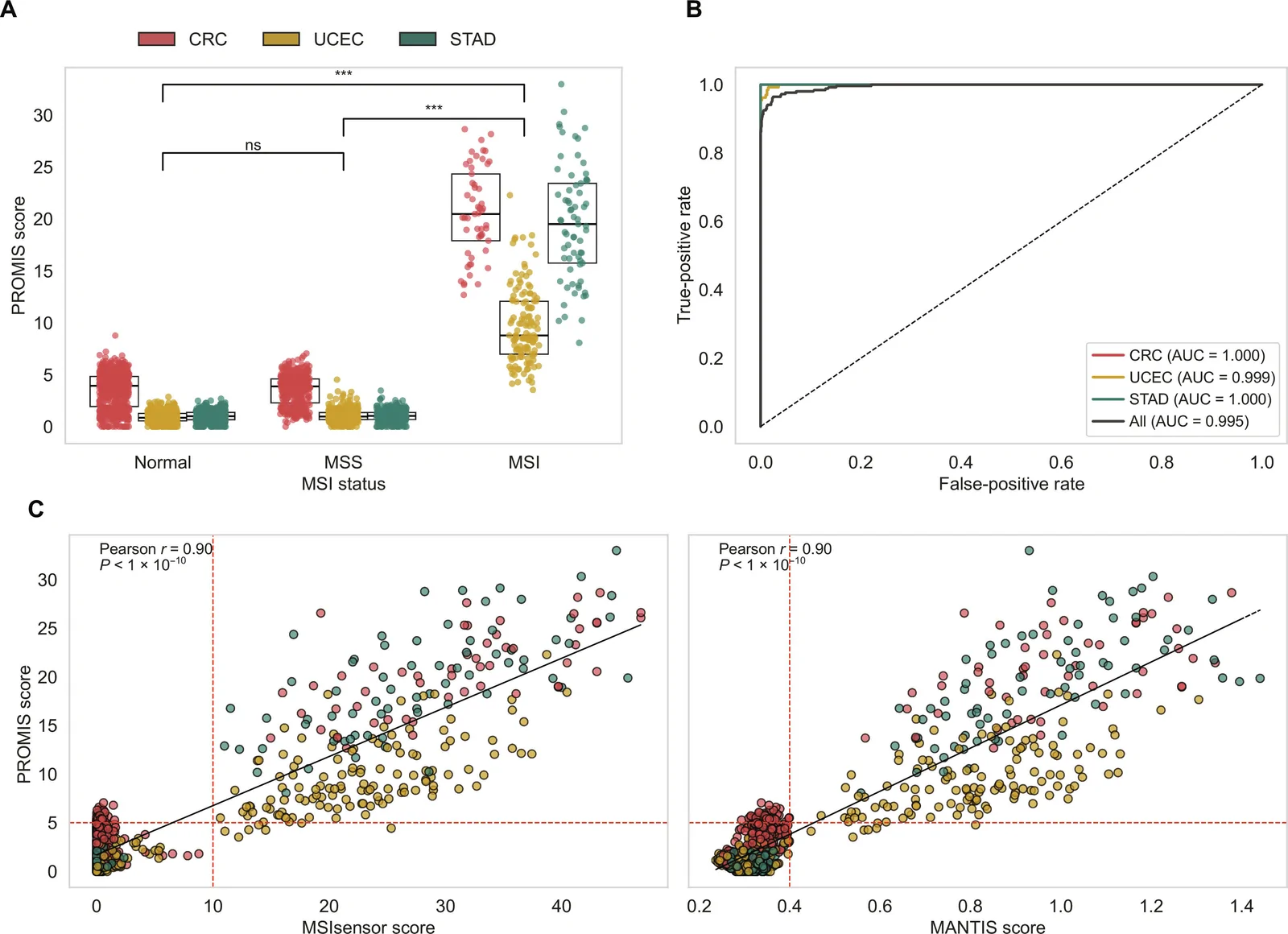
Performance across the evaluated sequencing contexts of the PROMIS (PROfiling of Microsatellite InStability) pipeline. (A) PROMIS scores by microsatellite instability (MSI) status. Each point represents a tumor sample, stratified by consensus MSI status (microsatellite-stable [MSS] or MSI) defined by concordant MSIsensor and MANTIS calls. Points are colored by cancer type (colorectal cancer [CRC], uterine corpus endometrial carcinoma [UCEC], and stomach adenocarcinoma [STAD]). Only samples with classifier agreement and mean coverage >30× are included. Corresponding normal tissue scores are shown in the left. (B) Receiver operating characteristic (ROC) curves for PROMIS performance in different cancer cohorts. (C) Correlation of PROMIS scores with established MSI classifiers. Left: Scatterplot of PROMIS *vs.* MSIsensor score; right: PROMIS *vs.* MANTIS score. Points are colored by cancer type. Red dashed lines indicate the classification thresholds for each method. Pearson correlation coefficients (*r*) and *P* values are shown. ***P < 0.001; ns, not significant.

Using a threshold of 5% unstable loci, sensitivity ranged from 95% in UCEC to 100% in CRC and STAD (overall sensitivity, 97%), whereas specificity reached 100% in UCEC and STAD but only 87% in CRC, consistent with the higher baseline microsatellite variability previously reported in microsatellite-stable (MSS) colorectal tumors [13] (Fig. 2A). Increasing the CRC threshold to 10% unstable loci resulted in 100% sensitivity and specificity. Across all 3 tumor types, PROMIS achieved an overall area under the receiver operating characteristic curve (AUC) of 0.995 (Fig. 2B). Overall performance was comparable to state-of-the-art MSI detection methods despite requiring neither matched normal samples nor pretrained machine learning models (Table 1) [5–8,14–16]. PROMIS scores strongly correlated with both MSIsensor (*r* = 0.904) and MANTIS (*r* = 0.901; both *P* < 10^−10^), demonstrating excellent concordance with established MSI callers (Fig. 2C). Among the few discordant cases, tumors classified as MSI by MANTIS but borderline by MSIsensor were generally classified as MSI by PROMIS, whereas tumors classified as MSS by MSIsensor and borderline by MANTIS remained MSS (Fig. S1). After exclusion of *POLE*-mutated tumors, PROMIS scores also correlated strongly with tumor mutational burden and total mutation counts, consistent with the hypermutated phenotype of MSI tumors (Fig. S2).

**Table 1.** Comparative AUC performance of MSI detection methods across 3 cancer types

Dataset	Method	Input	CRC	STAD	UCEC	Total
TCGA	PROMIS	T	1.000	1.000	0.999	0.995
TCGA	MANTIS	T–N	0.983	1.000	0.993	0.986
TCGA	MSIsensor	T–N	0.981	1.000	0.988	0.989
TCGA	mSINGS	T–PoN	0.594	0.711	0.634	0.594
TCGA	MSIsensor-pro	T–PoN	0.997	1.000	0.99	0.994
MSK-IMPACT	MiMSI	T–N	NA	NA	NA	0.972
MSK-IMPACT	MSIsensor	T–N	NA	NA	NA	0.907

Area under the receiver operating characteristic curve (AUC) values are shown for each method and dataset, stratified by cancer type: colorectal cancer (CRC), stomach adenocarcinoma (STAD), and uterine corpus endometrial carcinoma (UCEC), as well as the overall total. Methods are grouped by input requirements: tumor-only (T; PROMIS), matched tumor–normal pairs (T–N; MANTIS, MSIsensor, MiMSI, MSIsensor on Memorial Sloan Kettering-Integrated Mutation Profiling of Actionable Cancer Targets [MSK-IMPACT]), and tumor plus panel of normals (T–PoN; mSINGS, MSIsensor-pro). NA, not available for that cohort; MSI, microsatellite instability; PROMIS, PROfiling of Microsatellite InStability; TCGA, The Cancer Genome Atlas

To characterize the repeat-length alterations captured by PROMIS, we analyzed qualifying repeat-unit shifts in MSI tumors (Fig. S3). Single-repeat-unit shifts predominated across all 3 tumor types, accounting for 94% to 96% of events, with a modestly higher proportion in UCEC than in CRC (*P*_*adj*_ = 0.004) or STAD (*P*_*adj*_ = 1.4 × 10^−4^). Repeat contractions, i.e., a decrease in the length of a repeat sequence, likewise predominated (CRC 76.0%, UCEC 70.5%, STAD 78.4%) but were less frequent in UCEC (CRC *P*_*adj*_ = 0.02; STAD *P*_*adj*_ = 3.4 × 10^−5^).

**Fig. 3.**
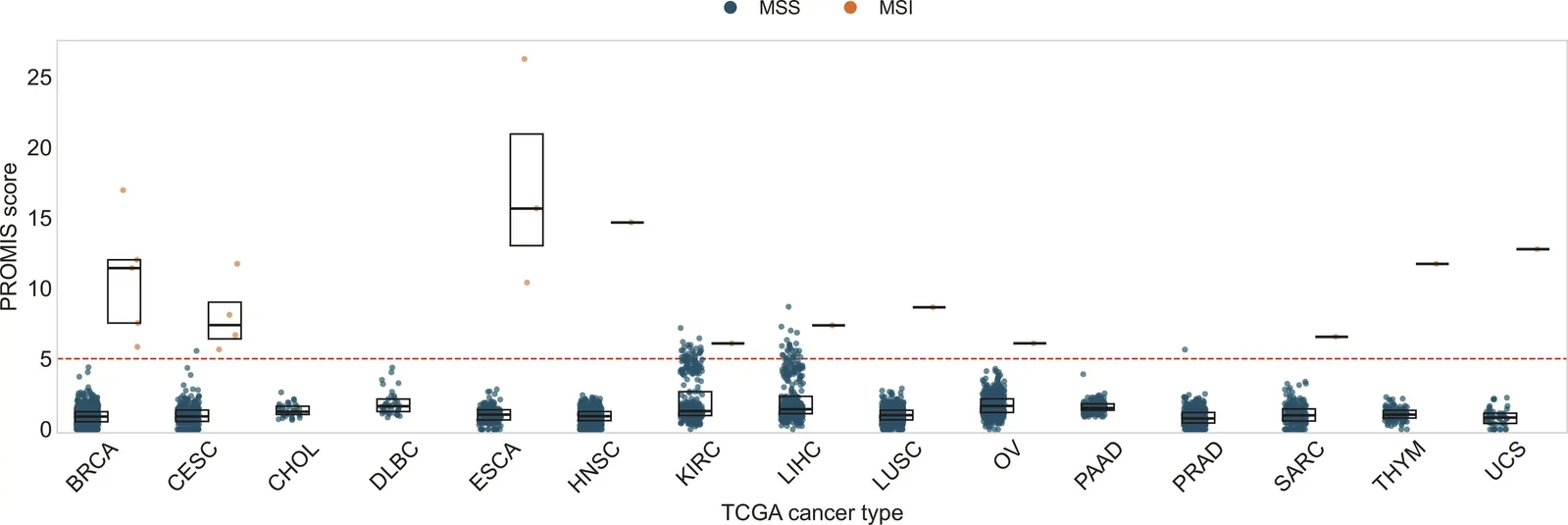
Pan-cancer distribution of PROMIS (PROfiling of Microsatellite InStability) scores across additional The Cancer Genome Atlas (TCGA) cohorts. PROMIS scores are shown for individual tumor samples stratified by cancer type and consensus microsatellite instability (MSI) status (microsatellite-stable [MSS] or MSI). Each point represents 1 tumor sample; boxes indicate the interquartile range, with the central line representing the median. Only samples with classifier agreement and mean coverage >30× are included. The analysis included all cancer types containing at least 1 concordant MSI case, together with cholangiocarcinoma (CHOL), diffuse large B-cell lymphoma (DLBC), pancreatic adenocarcinoma (PAAD) and prostate adenocarcinoma (PRAD), in which MSI has been reported but no concordant MSI cases were identified in the TCGA dataset. Horizontal dotted lines are visual guides for the PROMIS scores. BRCA, breast invasive carcinoma; CESC, cervical squamous cell carcinoma and endocervical adenocarcinoma; ESCA, esophageal carcinoma; HNSC, head and neck squamous cell carcinoma; KIRC; kidney renal clear cell carcinoma; LIHC, liver hepatocellular carcinoma; LUSC, lung squamous cell carcinoma; OV, ovarian serous cystadenocarcinoma; SARC, sarcoma; THYM, thymoma; UCS, uterine carcinosarcoma.

To further assess the pan-cancer applicability of PROMIS, we analyzed WES data from 15 additional TCGA cancer cohorts, including all cohorts with at least 1 MSI case, as well as pancreatic adenocarcinoma (PAAD) and prostate adenocarcinoma (PRAD), in which MSI has been reported but no MSI cases were present in the analyzed TCGA subset. All MSI tumors had PROMIS scores >5, whereas baseline PROMIS scores among MSS tumors varied across cancer types, with higher values observed in liver hepatocellular carcinoma (LIHC) and kidney renal clear cell carcinoma (KIRC) (Fig. 3).

PROMIS was further validated in an independent dataset of a multiregional metastatic cohort from database of genotypes and phenotypes (dbGaP) (accession phs001925.v1.p1) comprising 31 tumor samples and 7 matched normal samples from 6 patients with colorectal, gastric, duodenal, endometrial, and prostate cancers with MSI primary tumors [17]. PROMIS scores remained low in matched normal samples but varied substantially across tumor regions within individual patients, consistent with regional heterogeneity in MSI burden. PROMIS scores strongly correlated with matched MSIsensor results across tumor regions (*r* = 0.86), supporting robust performance in an independent dataset (Fig. S4).

### Robustness across genomic subsets and tumor fraction

**Fig. 4.**
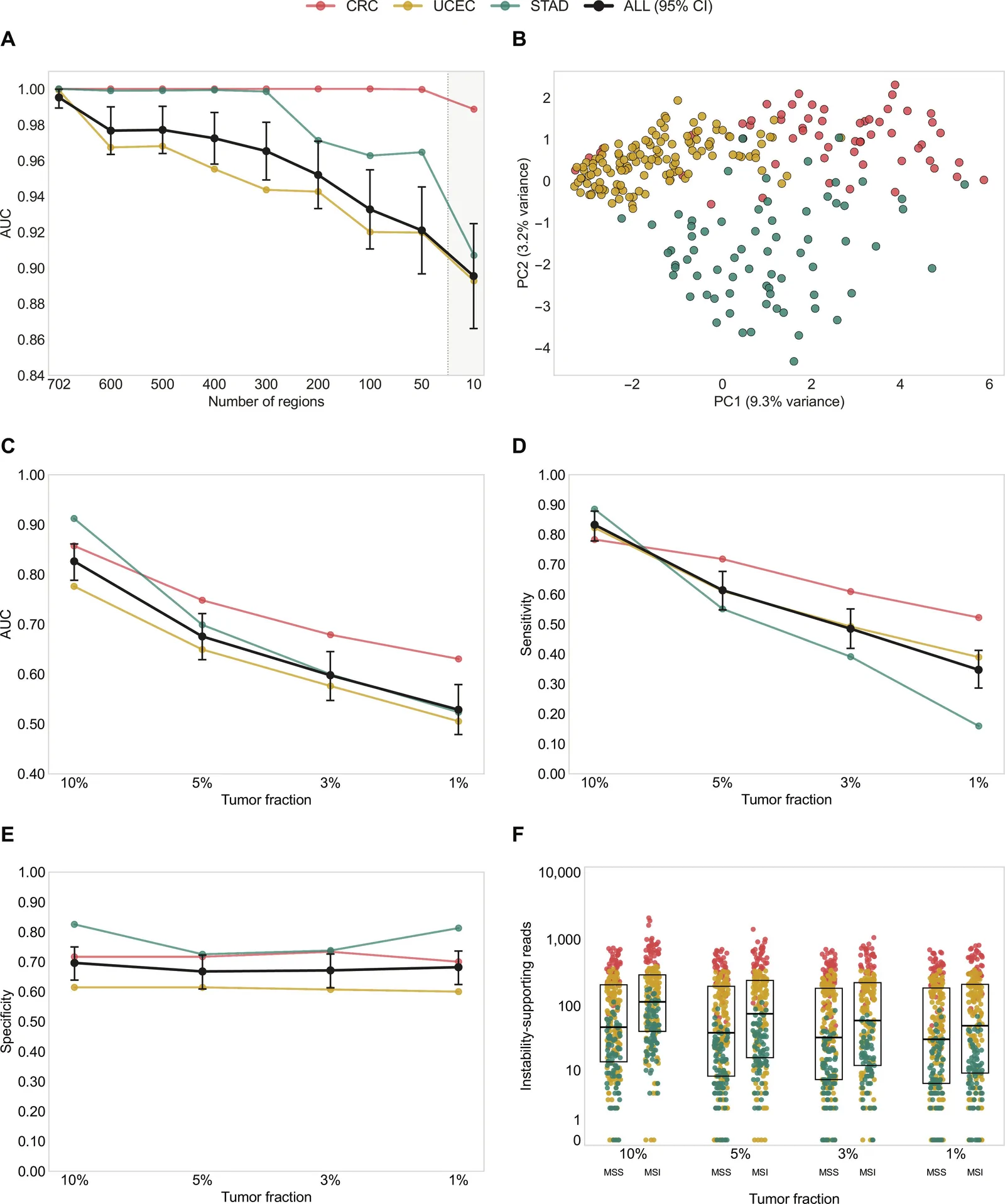
Performance of PROMIS (PROfiling of Microsatellite InStability) across genomic subsets and tumor fractions. (A) Area under the receiver operating characteristic curve (AUC) for PROMIS classification according to the number of microsatellite regions analyzed. Region sets were progressively reduced from 702 to 10 loci. Gray shading denotes exploratory conditions with fewer than 50 evaluated regions. (B) Principal component analysis of instability profiles across recurrent microsatellite loci in consensus microsatellite instability (MSI) tumors. Each point represents 1 tumor, and the variance explained by each component is indicated on the corresponding axis. (C to E) PROMIS performance across in silico tumor fractions of 10%, 5%, 3%, and 1% in 513 tumors (233 MSI and 280 microsatellite-stable [MSS]). (C) AUC, reflecting threshold-independent separation of MSI and MSS scores. (D) Sensitivity and (E) specificity at a PROMIS cutoff of 5 for stomach adenocarcinoma (STAD) and uterine corpus endometrial carcinoma (UCEC) and 10 for colorectal cancer (CRC). (F) Instability-supporting reads across tumor fractions. Each point represents 1 sample at the indicated fraction; MSS and MSI samples are shown in the left and right subcolumn, respectively. Boxes indicate the median and interquartile range, and read counts are displayed on a log-transformed scale. Colors denote cancer type (CRC, UCEC, and STAD), whereas black denotes pooled estimates (ALL). Error bars in panels (A) and (C) to (E) indicate 95% confidence intervals (CIs) for the pooled estimates.

To assess robustness across sequencing contexts, we evaluated progressively smaller nested subsets of the original 702 microsatellite loci in the TCGA cohort. With 50 loci, cancer-specific AUCs ranged from 0.92 to 1.0, with a pooled AUC of 0.92 (Fig. 4A). Reducing the analysis to 10 loci decreased the pooled AUC to 0.90, with the largest decline observed in UCEC and STAD. PROMIS therefore retained discriminatory capacity with substantially fewer loci, although these results support the use of at least 50 evaluable microsatellites for routine interpretation. Interestingly, principal component analysis revealed distinct tumor-type clustering of MSI tumors (Fig. 4B).

To assess performance across tumor fractions, we analyzed 513 tumors (233 MSI and 280 MSS; CRC, UCEC, and STAD) by spiking tumor-derived sequencing reads into the corresponding adjacent-normal sequencing data to generate simulated tumor fractions of 10%, 5%, 3%, and 1%. A common permissive locus-calling configuration revealed higher baseline microsatellite variability in CRC, consistent with the original tissue analysis (Fig. 2A), resulting in a higher median PROMIS score in CRC MSS samples (25.4) than in UCEC (4.2) or STAD (3.6) and a CRC specificity of 0.22 (Fig. S5C). Introducing a more stringent CRC-specific threshold effectively reduced this background, increasing CRC specificity to 0.70 to 0.73 across tumor fractions. Sensitivity was highest at 10% tumor fraction (0.78) and progressively decreased to 0.52 at 1%, consistent with the diminishing tumor-derived signal. Across all tumor types, PROMIS retained good discrimination at 10% tumor fraction, with a pooled AUC of 0.83 and sensitivity of 0.83. As expected, performance progressively decreased at lower tumor fractions, reaching an AUC of 0.53 and sensitivity of 0.35 at 1%, while specificity remained comparatively stable at 0.67 to 0.70. This increased CRC specificity to 0.70 to 0.73, with sensitivity decreasing from 0.78 at 10% to 0.52 at 1%. The pooled AUC declined from 0.83 at 10% to 0.53 at 1% (Fig. 4C). Pooled sensitivity decreased from 0.83 to 0.35, whereas specificity remained between 0.67 and 0.70 (Fig. 4D and E).

Analysis of intermediate PROMIS outputs further illustrated the progressive loss of MSI-derived signal with decreasing tumor fraction. In MSI tumors, the median number of unstable loci declined from 22 at 10% to 13, 11, and 9 at 5%, 3%, and 1%, respectively; the corresponding median numbers of instability-supporting reads were 115, 76, 60, and 50. In contrast, the MSS background remained comparatively stable, with median unstable-locus counts of 9, 7, 7, and 6 and supporting-read counts of 48, 39, 33, and 31 across the same fractions (Fig. S6). Despite CRC-specific optimization, the median MSS PROMIS score at 1% remained higher in CRC (9.11) than in UCEC (4.35) or STAD (3.31), indicating an increasing contribution of tumor-type-specific baseline microsatellite variability at very low tumor fractions.

Consistent with the original tissue analysis, 1-bp shifts predominated across MSI tumors and tumor fractions, accounting for 89.8% of qualifying deviating reads, whereas 2-bp and ≥3-bp shifts accounted for 8.9% and 1.3%, respectively (Fig. S6F).

### Application to cfDNA and targeted panels

Detection of MSI in cfDNA is clinically relevant but remains challenging because of low tumor fractions, variable circulating tumor DNA (ctDNA) shedding, and the limited genomic coverage of targeted sequencing assays.

**Fig. 5.**
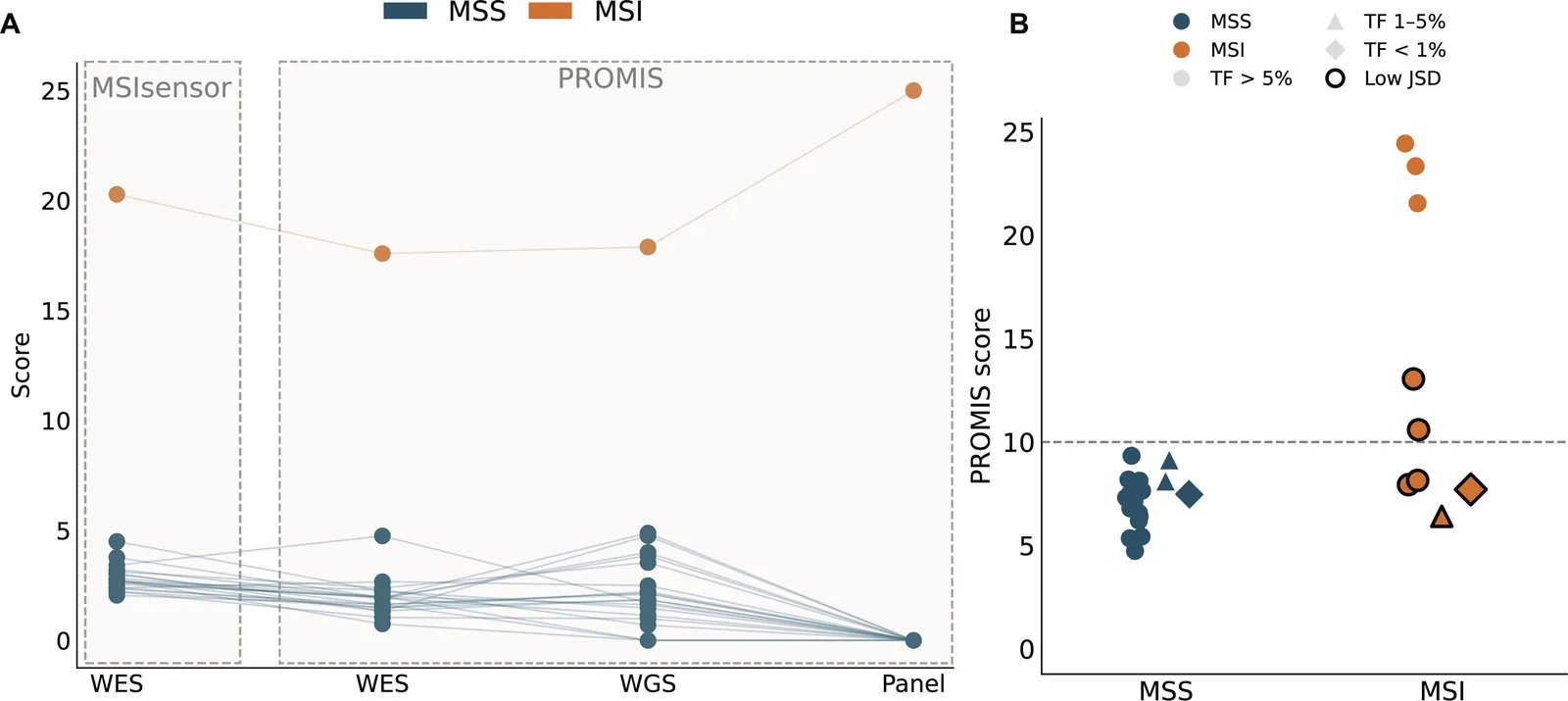
PROMIS (PROfiling of Microsatellite InStability) performance in liquid biopsy assays. (A) Comparison of PROMIS scores with MSIsensor in prostate cancer cell-free DNA (whole-exome sequencing [WES] and 20× whole-genome sequencing [WGS]) and targeted panel (18 genes, QIAseq-HRD). Each point represents a patient sample, colored by consensus microsatellite instability (MSI) status (microsatellite-stable [MSS] or MSI). Lines connect scores across methods for the same sample. Gray dashed boxes highlight MSIsensor (left) and PROMIS (right) groups of methods. (B) PROMIS scores in TSO500 circulating tumor DNA samples stratified by reported MSI status. Each point represents a patient sample, and scores are expressed as the percentage of unstable microsatellite regions. Circles, triangles, and diamonds denote samples withtumor fraction (TF) >5%, TF of 1% to 5%, and TF <1%, respectively. Black outlines indicate samples with low Jensen–Shannon distance (JSD) values (0.1 to 0.6).

To evaluate the applicability of PROMIS in this setting, we validated the approach using in-house cfDNA sequencing datasets generated across multiple assay types. In prostate cancer cfDNA, PROMIS was applied to WES, WGS, and an 18-gene targeted panel [17]. Among 21 patients, PROMIS consistently detected 1 MSI-positive case across all assay types, with instability fractions concordant with reference classifications (Fig. 5A).

PROMIS was further evaluated in cfDNA samples of cancer patients (CRC, *n* = 32; prostate cancer, *n* = 26; pancreatic cancer, *n* = 1; esophageal cancer, *n* = 1; multiple tumors, *n* = 1) referred to our Molecular Tumor Board. Liquid profiling was performed using the TruSight Oncology 500 ctDNA assay (TSO500, Illumina). A total of 9/61 samples were called as MSI by the TruSight Oncology 500 ctDNA algorithm, and PROMIS scores showed separation between MSI and MSS cases (Fig. 5B). As observed in the TCGA cohorts, CRC samples exhibited higher baseline instability, supporting a tumor-specific threshold of 10% unstable loci. Borderline or false-negative cases were largely confined to samples with tumor fractions <5%. In addition, excluding loci classified as unstable in ≥30% of samples substantially reduced recurrent background signal while largely preserving MSI scores (Fig. S7), supporting the feasibility of PROMIS for targeted cfDNA sequencing while highlighting the importance of background correction in high-coverage panels. In such cases, reduced Jensen–Shannon distance values used by TSO500 pipeline may reflect biological signal dilution or sample-specific germline microsatellite variation not represented in the baseline panel.

Finally, we performed an exploratory evaluation of 9 Ion Torrent samples (MSS, *n* = 7; MSI, *n* = 2). PROMIS did not achieve robust discrimination, with substantial overlap between MSS (79.2 to 90.6) and MSI-H (64.8 to 81.4) scores (Fig. S8), consistent with the known limitations of Ion Torrent for accurate repeat-length determination.

## Discussion

Accurate detection of MSI across diverse sequencing contexts remains a central challenge for precision oncology. Here, we developed and validated PROMIS, a reference-free MSI detection method based on repeat-length distributions that does not require matched normal samples or pretrained models. All analytical steps produce traceable intermediate outputs, supporting reproducibility, interpretability, and clinical auditability. PROMIS achieved performance comparable to established MSI callers across TCGA cohorts [5–8] and generalized to additional tumor types, independent datasets, and Illumina-based cfDNA assays. It retained robust discrimination across sequencing contexts, while the dilution experiments quantified the decline in performance at low tumor fractions. Together, these findings demonstrate that MSI can be robustly inferred from the intrinsic repeat-length distributions of individual tumor samples. Unlike binary classifiers, PROMIS also provides continuous instability scores, enabling more nuanced characterization of the MSI phenotype.

The predominance of single-repeat-unit shifts across CRC, UCEC, and STAD suggests a broadly conserved mechanism of microsatellite slippage, whereas the lower proportion of contractions in UCEC indicates modest tumor-type-specific differences in shift direction (Fig. S3). Although the repeat-length distributions of detected unstable loci were largely similar across tumor types, subtle shifts supported by few reads are more susceptible to sequencing noise and polymerase chain reaction-associated stutter and may therefore fail to meet the locus-level instability criteria. Consequently, genuine MSI may result in fewer unstable loci and lower PROMIS scores, particularly in UCEC, where MSI tumors have been reported to exhibit smaller repeat-length shifts than CRC [18,19]. Consistent with this interpretation, recurrently unstable microsatellite loci differed between tumor types, as reflected by tumor-type clustering in principal component space and the expanded pan-cancer TCGA analysis, indicating that the genomic landscape of MSI is, at least partly, tumor-type-specific (Fig. 4B).

These differences have practical implications for MSI classification. In the expanded TCGA analysis, all concordant MSI tumors had PROMIS scores >5, supporting this value as a provisional pan-cancer threshold. However, higher baseline instability in CRC supported a tumor-specific threshold >10, while elevated baseline scores in subsets of MSS KIRC and LIHC suggest that additional tumor-specific calibration may also be required for these cancers. Similar tumor-specific thresholds have been reported for other next-generation sequencing-based MSI assays [20], underscoring the importance of cancer-specific calibration. Because too few MSI-positive KIRC and LIHC cases were available, robust tumor-specific cutoffs could not be derived. Larger MSI-enriched cohorts will therefore be required to establish cancer-specific thresholds. Until then, PROMIS scores between 5 and 10 should be interpreted in the context of tumor type, sequencing quality, read depth, and the number of evaluable loci, with borderline results confirmed using an orthogonal MSI assay.

Our analyses further demonstrated that MSI detection can be performed reliably even when only a limited number of microsatellite loci are available, as is typical for targeted sequencing panels. Subsampling experiments showed stable performance despite substantial reductions in locus number, indicating that PROMIS captures global instability patterns rather than relying on individual marker loci. The observed tumor-type-specific differences in informative loci and baseline instability support quantitative scoring rather than fixed marker panels, facilitating application across clinical sequencing workflows with diverse panel designs. More broadly, genome-scale short tandem repeat resources demonstrate substantial diversity in microsatellite content across human haplotypes and may support the future refinement of assay-specific locus selection [21].

Accurate MSI detection in cfDNA is becoming increasingly important as ctDNA-based comprehensive genomic profiling is increasingly used when tissue is unavailable or insufficient or repeat biopsy is impractical. Furthermore, in tumor types where MSI is rare, routine MSI testing is often omitted because of its low expected yield, whereas comprehensive ctDNA profiling is increasingly performed to identify other actionable alterations. PROMIS enables MSI status to be inferred from these existing sequencing data, potentially identifying the rare patients eligible for ICI therapy without requiring an additional dedicated assay. Consistent with this concept, PROMIS accurately identified the single MSI case across WES, WGS, and targeted sequencing in an in-house prostate-cancer cfDNA cohort [17] and further discriminated MSI from MSS cases in 61 TSO500-profiled cfDNA samples, supporting its applicability across targeted cfDNA sequencing workflows.

However, MSI detection in cfDNA remains challenging because of low tumor fractions, variable ctDNA shedding, and limited genomic coverage [22,23]. Our dilution experiments showed that decreasing tumor-derived signal progressively reduced MSI-MSS discrimination and, importantly, highlighted the influence of tumor-type-specific background. This could not be addressed by universally relaxing locus-calling criteria at lower tumor fractions; instead, tumor-type-aware criteria were required, particularly for CRC. Thus, tumor type affects not only the PROMIS score required for classification but also the locus-level evidence needed to distinguish true instability from background. Based on these findings, we recommend 10% as a conservative minimum tumor fraction for reliable interpretation with the current workflow, while results below this threshold should be considered exploratory and confirmed using an orthogonal MSI assay.

Beyond MSI classification, the modular architecture of PROMIS facilitates adaptation to new sequencing panels and enables biologically informed analyses of locus-specific instability patterns. For example, TA-repeat instability associated with WRN helicase dependency has emerged as a potential therapeutic biomarker [24,25]. Incorporating gene- and motif-level annotations may therefore extend PROMIS from binary MSI classification toward biologically informed patient stratification without requiring fundamental methodological changes.

Taken together, by providing transparent locus-level instability calls together with visualizable repeat-length distributions, PROMIS enables direct interrogation of the underlying biological signal, facilitating clinical auditing, molecular tumor board review, and distinction between biological instability and technical artifacts. These features make PROMIS well suited for deriving MSI status from sequencing data generated for broader comprehensive genomic profiling, without requiring an additional dedicated MSI assay.

Nevertheless, several limitations should be considered. First, although tumor-type-aware locus-calling criteria improved apparent performance in CRC, UCEC, and STAD, they were derived and evaluated in the same retrospective in silico dilution cohort and therefore require independent validation. The limited number of MSI-positive KIRC and LIHC cases precluded comparable tumor-specific calibration. Larger MSI-enriched cohorts are needed to determine whether distinct criteria are also required for these and other cancer types. Second, the present study establishes the analytical validity of PROMIS but not its predictive clinical validity. Although PROMIS showed concordance with orthogonal MSI assays, its association with ICI response and clinical outcomes was not evaluated. Prospective studies with linked treatment outcomes will therefore be required before PROMIS can be used to guide treatment decisions, particularly in tumor types in which MSI is rare.

Third, validation was performed primarily on Illumina-derived sequencing data. PROMIS did not achieve robust discrimination in the exploratory Ion Torrent cohort, consistent with the limited accuracy of homopolymer length determination on semiconductor sequencing platforms [26]. Accordingly, the current implementation should be considered applicable only to Illumina-based sequencing workflows.

Finally, the in silico dilution experiments showed that PROMIS performance declined at low tumor fractions and varied substantially among tumor types. Although tumor-type-aware criteria reduced background instability, these experiments do not establish performance under real-world cfDNA conditions, where tumor shedding, fragment length, sequencing depth, and preanalytical factors may differ. Validation in larger, disease-specific cfDNA cohorts spanning a broad range of circulating tumor fractions is therefore required.

In summary, PROMIS provides a reference-free, interpretable, and assay-adaptable approach for MSI detection across tissue and liquid-biopsy sequencing data. Rather than replacing established MSI companion diagnostics, PROMIS is designed to complement sequencing workflows that lack validated MSI calling. While several commercial comprehensive genomic profiling assays incorporate MSI assessment, many custom targeted panels and institutional WES/WGS workflows do not. In these settings, PROMIS enables MSI detection directly from existing sequencing data without requiring an additional dedicated assay. By combining assay flexibility with locus-level interpretability, PROMIS provides a strong foundation for further analytical optimization and prospective clinical validation and may facilitate broader implementation of MSI-guided precision oncology.

## Methods

### Data sources and MSI reference labels

WES data for CRC (COAD/READ), UCEC, and STAD were obtained from TCGA (RRID:SCR_003193). Aligned and preprocessed BAM files (hg38) were downloaded from the Genomic Data Commons (GDC, RRID:SCR_014514). Samples with mean coverage below 30× were excluded from analysis.

Reference MSI classifications were derived from published TCGA consensus annotations generated using MSIsensor (RRID:SCR_006418) and MANTIS, applying their standard thresholds of 10 and 0.4, respectively [5,6]. Only samples with concordant calls between both tools were used as benchmark reference labels. Discrepant cases were excluded from the validation set. Only loci with at least 50 evaluable reads were considered. A locus was classified as unstable if at least 5 reads deviated from the dominant stable allele and collectively accounted for at least 10% of evaluable reads, with each individual deviating repeat length supported by a read count equivalent to at least 20% of the read count supporting the dominant stable allele.

Repeat-length shifts were derived from PROMIS locus-level outputs and measured from the nearest stable-allele boundary, with negative and positive values representing contractions and expansions, respectively. Shifts were normalized by motif length, and only changes corresponding to an integer number of repeat units were retained. Contraction and exact one-repeat-unit proportions were calculated per tumor and summarized using equal tumor weighting. Confidence intervals were obtained from 10,000 tumor-level bootstrap resamples. Cancer types were compared using 2-sided Mann–Whitney U tests with Benjamini–Hochberg correction.

To extend the evaluation of PROMIS across additional tumor types, WES data from 15 TCGA cohorts were analyzed: breast invasive carcinoma, cervical squamous cell carcinoma and endocervical adenocarcinoma, cholangiocarcinoma, lymphoid neoplasm diffuse large B-cell lymphoma, esophageal carcinoma, head and neck squamous cell carcinoma, KIRC, LIHC, lung squamous cell carcinoma, ovarian serous cystadenocarcinoma, PAAD, PRAD, sarcoma, thymoma, and uterine carcinosarcoma. Cohort selection included all available additional cancer types containing at least 1 MSI case according to the reference annotations, together with selected tumor types in which MSI is uncommon, including PAAD and PRAD.

For validation in a multiregional metastatic cohort, we analyzed WES data from dbGaP study phs001925 [17]. The cohort comprised 31 tumor samples and 7 matched normal samples from 6 patients with CRC, UCEC, PRAD, STAD and duodenal with MSI primary tumors. Sequence Read Archive (SRA) files were retrieved, and FASTQ files were generated from the SRA archives using National Center for Biotechnology Information SRA Toolkit v3.4.1 (RRID:SCR_024350). Datasets were aligned and processed according to the Genome Analysis Toolkit (GATK) Best Practices workflow (RRID:SCR_001876), including alignment with BWA-MEM2 (RRID:SCR_022192) [25], sorting, duplicate marking, and base quality recalibration, using the hg38 reference genome. PROMIS was then applied to the resulting aligned sequencing data using the same workflow and scoring framework as for the other tissue cohorts. MSIsensor scores were derived from matched tumor-normal WES analyses of the same cohort.

For the exploratory assessment of semiconductor sequencing data, we analyzed 9 MSI annotated Ion Torrent tissue samples (7 MSS and 2 MSI). BAM files generated with the Oncomine Comprehensive Assay on the Ion Torrent platform and aligned to hg19 were processed with PROMIS using the same parameters as in the other tissue analyses.

For cfDNA analyses, prostate cancer samples were analyzed using WES, WGS, and targeted amplicon panel sequencing [17]. All datasets were aligned and processed according to the GATK Best Practices workflow (RRID:SCR_001876), including alignment with BWA-MEM2 (RRID:SCR_022192) [27], sorting, duplicate marking, and base quality recalibration, using the hg38 reference genome. MSI status for these samples was determined from matched tumor-normal MSIsensor calls on the WES data.

cfDNA samples profiled using the Illumina TruSight Oncology 500 ctDNA assay were processed with the DRAGEN TSO500 ctDNA analysis workflow on the Illumina DRAGEN Bio-IT Platform. The assay targets 523 cancer-associated genes and reports tumor mutational burden and MSI metrics. The tumor-only MSI module compares microsatellite repeat-length distributions in each sample with distributions derived from baseline normal samples using Jensen–Shannon distance and statistical testing. Evidence from unstable sites is then aggregated to generate the sample-level MSI metric and reported classification. Depending on data availability, PROMIS was applied to BAM or CRAM files aligned to the hg19 reference genome. The MSI status reported by the TSO500/DRAGEN workflow was used as the reference label for comparison with PROMIS scores. For both cfDNA analyses, PROMIS used the same locus-calling thresholds as in the main tissue analysis.

### Workflow overview

PROMIS operates on aligned BAM/CRAM files and a reference set of microsatellite loci defined in the corresponding genome build. The workflow proceeds through sequential stages, each focused on a specific analytical task. All parameters like base and mapping quality or MSI calling thresholds can be readily adjusted through a configuration file.

#### Read extraction and filtering

Sequencing reads overlapping predefined microsatellite loci are identified using coordinate-based queries. Only reads that span the full repeat region with at least 4 bp of flanking sequence on each side are retained. Quality control filters exclude reads with mapping quality below 40 or mean base quality below 20 within the repeat tract. Reads containing soft-clipped alignments, supplementary mappings, or mismatched repeat motifs are also discarded to prevent artifacts from misalignments or sequencing errors. The retained reads are used to reconstruct the observed repeat length per locus, producing a read-level repeat-length profile for each sample.

#### Repeat-length quantification

For each microsatellite locus, the distribution of observed repeat lengths across all high-quality reads is computed. Stable loci typically display 1 or 2 dominant alleles (corresponding to homozygous or heterozygous germline configurations), while unstable loci exhibit additional repeat-length variants reflecting somatic indel events. Loci with fewer than 50 total reads are flagged as low-confidence and excluded from downstream modeling.

#### Instability modeling and locus classification

For each locus *l*, the likelihood of observing a repeat length *x* across *n* reads is expressed as:

$$p\left(x|l\right)=\sum_{k=1}^{K}\pi_{k}𝒩\left(x|\mu_{k},\sigma_{k}^{2}\right)$$ (1)

where *x* represents an individual repeat length observation (in base pairs); *K* is the number of mixture components (ranging from 1 to 4), representing distinct allelic populations; *πk* is the mixing coefficient (relative proportion) of reads assigned to component *k*, with *∑πk* = 1 and 0 ≤ *πk* ≤ 1; *μk* is the mean repeat length of component *k* (in base pairs); *(σk)*^*2*^ is the variance of component *k*, capturing the spread of repeat lengths around the mean; and *𝒩(x | μ*_*k*_*, (σ*_*k*_*)*^*2*^*)* is the Gaussian probability density function for component *k.*

Although repeat lengths are discrete integers, a continuous Gaussian approximation provides a stable estimate of distinct allelic modes under moderate coverage conditions.

Model parameters *Θ = π₁...π_K, μ₁...μ_K, σ₁*^*2*^*...σ_K*^*2*^*}* were estimated using the expectation–maximization algorithm implemented in scikit-learn’s GaussianMixture class (RRID:SCR_002577). To ensure numerical stability and prevent singular covariance matrices in cases of limited variance, we applied regularization by adding a small constant to the diagonal of covariance matrices:

$$\Sigma_{k}^{\prime }=\Sigma_{k}+\lambda \cdot I$$ (2)

where *Σk* is the covariance matrix for component *k*; *λ = 1 × 10*^*−2*^ is the regularization coefficient (reg_covar parameter); ***I*** is the identity matrix; and *Σ*_*k′*_ is the regularized covariance matrix used in model fitting.

Each model was initialized 5 times (*n*_init_ = 5) with different random starting values, and the solution achieving the highest log-likelihood was retained to mitigate local optima in the expectation–maximization algorithm.

The optimal number of mixture components *K* is selected by minimizing the Bayesian Information Criterion (BIC):

$$BIC=-2ln\left(\hat{L}\right)+pln\left(n\right)$$ (3)

where $\;\hat{L\;}$is the maximized likelihood of the fitted model, representing how well the model explains the observed data; *p* is the number of free parameters in the model (for a GMM with *K* components: *p* = *K* − 1 mixing weights + *K* means + *K* variances = 3*K* − 1); and *n* is the number of reads supporting the locus.

To prevent overfitting and distinguish biologically meaningful germline heterozygosity from technical noise or somatic instability, a secondary allele (μ₂) was retained only if all of the following criteria were satisfied:

1. Adequate representation: $\pi_{2}\geq f_{\min}$ where *f*_*min*_ = 0.25 (minimum fraction of reads, default parameter)
2. Balanced allelic ratio: $|\frac{\pi_{2}}{\pi_{1}}-1|\leq \tau$ where *τ* = 0.05 (balance tolerance, default parameter)

These stringent criteria ensure that secondary components represent genuine heterozygous germline variants (expected ratio ≈ 1:1) rather than low-level somatic mutations or sequencing artifacts. A locus is classified as heterozygous when both alleles meet these thresholds; otherwise, it is treated as homozygous with a single stable length.

#### Locus-level instability scoring and sample-level MSI computation

Following mixture model fitting, each microsatellite locus was evaluated for evidence of instability based on its repeat-length distribution. A locus was designated as unstable when a sufficient fraction of reads deviated from the inferred stable allelic modes by at least 1 repeat unit. Deviations were quantified using 2 complementary criteria: a minimum number of deviating reads and a minimum percentage of the total reads supporting an alternate allele length. By default, loci with fewer than 50 total reads were excluded from analysis to minimize stochastic effects at low coverage, and a locus was called unstable if at least 5 deviating reads or ≥1% of all reads supported a distinct allele length. All thresholds, including minimum read depth, minimum deviation counts, and percentage cutoffs, are user-configurable within the PROMIS configuration file.

For each sample, PROMIS aggregates locus-level classifications to compute a global instability score, defined as the fraction of evaluated loci classified as unstable. This continuous MSI score reflects the overall burden of MSI within the tumor and can be readily converted to a binary MSI or MSS status by applying a user-defined cutoff.

The pipeline outputs comprehensive summary tables containing per-locus statistics (lengths of deviating reads, number of deviating reads, and instability flag) and sample-level metrics, allowing both automated classification and manual inspection of borderline cases.

#### Microsatellite locus definition and preprocessing

For tissue and prostate cfDNA analyses, PROMIS used a curated set of 702 high-quality microsatellite loci derived from the MOSAIC reference dataset [12], encompassing mono-, di-, tri-, and tetranucleotide repeats across exonic and clinically relevant regions.

For assay-specific targeted datasets, including the TSO500 ctDNA cohort and Ion Torrent cohort, PROMIS can generate a custom microsatellite locus set by identifying repeat loci present in the relevant genome build and covered by the sequencing assay. This assay-specific preprocessing step was used for the TSO500 data, which were aligned to hg19, ensuring that PROMIS scoring was based only on microsatellite loci represented in the targeted assay.

In all analyses, loci with insufficient read depth or mapping quality were excluded from downstream modeling. This preprocessing framework enables PROMIS to adapt dynamically to WGS, WES, and targeted panels without requiring retraining or recalibration.

### Assessment of number of regions needed for reliable calls

To evaluate the impact of locus number on MSI classification accuracy, all microsatellite regions were ranked according to their discriminatory power, callability, and background stability. For each locus, receiver operating characteristic AUC values were computed using consensus MSI labels from MSIsensor and MANTIS, together with callability rates (coverage >30×) and instability frequency in MSS samples. A composite score integrating these metrics was bootstrapped 100 times to obtain mean stability ranks, and loci were sorted accordingly. Nested subsets containing the top 600, 500, 400, 300, 200, 100, 50, and 10 loci were then used to recompute PROMIS MSI scores for all samples. Ninety-five percent confidence intervals for the pooled AUC estimates were calculated using the Hanley–McNeil variance estimator.

### Simulation of tumor fraction

To evaluate PROMIS performance at varying tumor fractions, in silico spike-in dilution series were generated by adding randomly selected read pairs from each tumor BAM with reads from the corresponding matched-normal BAM. Because the tumor BAMs already contained both tumor-derived and nontumor reads, the proportion of reads sampled from each tumor BAM was adjusted for its initial ABSOLUTE purity. Original tumor purity estimates were obtained from ABSOLUTE values (RRID:SCR_005198) [28], provided in the TCGA metadata. For each tumor, synthetic BAM files were created at 10%, 5%, 3%, and 1% tumor fractions. Mixing was performed in a read-pair-aware manner, preserving original pairing, and read-group metadata.

The final cohort comprised 233 MSI and 280 MSS tumors, including 106 CRC tumors (46 MSI and 60 MSS), 258 UCEC tumors (118 MSI and 140 MSS), and 149 STAD tumors (69 MSI and 80 MSS).

For each synthetic BAM, loci were required to have at least 50 evaluable reads. To accommodate the lower tumor fractions, a single, less stringent locus-calling configuration was applied across the entire dilution series. A locus was classified as unstable when at least 2 reads deviated from the inferred stable allele, the qualifying deviating reads represented at least 1% of the reads at that locus, and each individual deviating repeat-length class was supported by a read count corresponding to at least 3% of the dominant stable-allele count. Because this configuration produced substantially greater background instability in CRC, the final tumor-type-aware analysis used a fixed configuration of at least 6 qualifying deviating reads representing at least 1% of evaluable reads, with each deviating length class supported by at least 3% of the dominant stable-allele count. The PROMIS score was calculated as the percentage of evaluable loci classified as unstable.

Receiver operating characteristic AUC was used to assess threshold-independent discrimination between MSI and MSS tumors. Sensitivity and specificity were evaluated at PROMIS cutoffs of >10 for CRC and >5 for UCEC and STAD. Because these cutoffs differed, PROMIS scores were normalized to the applicable cancer-specific cutoff before calculating the pooled AUC. Ninety-five percent confidence intervals for AUC were derived from 10,000 sample-level bootstrap replicates stratified by MSI status. Exact binomial Clopper–Pearson confidence intervals were calculated for sensitivity and specificity.

## Ethical Approval

TCGA data were obtained from TCGA via the Genomic Data Commons and used in accordance with TCGA data use policies; all patients provided informed consent as part of the TCGA program.

The prostate cancer cfDNA study was approved by the Ethics Committee of the Medical University of Graz, Austria (approval number: EK Nr ex 21-228). All cfDNA participants provided written informed consent to participate, including collection of blood specimens for plasma genotyping and germline testing and use of associated clinical data for research. The study was conducted in accordance with the Declaration of Helsinki.

## Data Availability

TCGA data used in this study were obtained through the Genomic Data Commons (GDC) and are available to qualified researchers through the GDC and associated dbGaP-controlled-access mechanisms, in accordance with TCGA data-use policies. Data from the independent multiregional metastatic cohort are available through dbGaP under accession phs001925.v1.p1, subject to dbGaP authorization and the applicable data-use restrictions. Prostate cancer cfDNA sequencing data from the PROMIS cohort have been deposited in the European Genome-phenome Archive (EGA) under accession EGAS00001008190. Individual-level TSO500 cfDNA and Ion Torrent sequencing data are not publicly available because they were generated as part of clinical care and their public release is restricted by patient-consent, privacy, institutional, and data-protection requirements. Deidentified derived data may be made available by the corresponding author upon reasonable request, subject to institutional approval and applicable legal and ethical restrictions. The PROMIS pipeline is available at GitHub (https://github.com/vlachosg37/promis). The repository contains the core workflow, configuration templates, curated microsatellite locus sets, example input files, and Conda environment definitions required to reproduce the analyses presented in this study. Documentation in the repository describes supported input data types required preprocessing steps, and typical usage scenarios, including cfDNA applications.
