# Supplementary material for: Reference-Free Microsatellite Instability Detection from Tumor Sequencing Using Intrasample Variability Modeling
Source: Comput Struct Biotechnol J. 2026 Sep 9;35(1):0219. doi: 10.34133/csbj.0219 (PMC13554417; doi:10.34133/csbj.0219)
Supplement: Supplementary file 1 [file csbj.0219.f1.docx]

**Reference-free microsatellite instability detection from tumor sequencing using intrasample variability modeling**

**Running title: Tumor-only detection of microsatellite instability**

Georgios Vlachos^1,2^, Tina Moser^1^, Mitesh Patel^2^, James R. White^3^, Carina Pischler^1^, Lisa Glawitsch^1^, Thomas Bauernhofer^4^, Philipp Jost^4^, Leo Edlinger^4^**,** Karl Kashofer^5^, Jochen B. Geigl^1^, Luis A. Diaz Jr.^2^ and Ellen Heitzer^1*^

^1^Institute of Human Genetics, Diagnostic & Research Center for Molecular BioMedicine, Medical University of Graz, Austria; ^2^Division of Solid Tumor Oncology, Memorial Sloan Kettering Cancer Center, New York, NY, USA; ^3^Resphera Biosciences, Baltimore, MD, USA; ^4^Division of Oncology, Department of Internal Medicine, Medical University of Graz, Graz, Austria; ^5^Diagnostic and Research Institute of Pathology, Medical University Graz, Graz, Austria

*Correspondence to: [ellen.heitzer@medunigraz.at](mailto:ellen.heitzer@medunigraz.at)

Contents

[Supplementary Figure 1. Scatterplot of MSIsensor and MANTIS scores for TCGA colorectal (CRC), endometrial (UCEC), and gastric (STAD) tumors showing cases with discordant classifications between the two conventional MSI callers.	4](#_Toc238117720)

[Supplementary Figure 2. Scatterplots illustrating the relationship between tumor mutational burden and PROMIS score across TCGA colorectal (CRC), endometrial (UCEC), and gastric (STAD) cancers.	5](#_Toc238117721)

[Supplementary Figure 3. Repeat-unit distributions of microsatellite length changes across TCGA cancer types.	6](#_Toc238117723)

[Supplementary Figure 4: PROMIS score versus matched tumor-normal MSIsensor score in the multiregional metastatic phs001925.v1.p1 cohort.	7](#_Toc238117724)

[Supplementary Figure 5: Performance of PROMIS across genomic subsets and tumor fractions with the common relaxed configuration.	8](#_Toc238117725)

[Supplementary Figure 6: Detailed PROMIS metrics across decreasing tumor fractions.	9](#_Toc238117726)

[Supplementary Figure 7. Effect of recurrent unstable region filtering on PROMIS scores in TSO500 cfDNA samples.	10](#_Toc238117728)

[Supplementary Figure 8. PROMIS scores in the exploratory Ion Torrent cohort.	11](#_Toc238117729)

Supplementary Figure 1. Scatterplot of MSIsensor and MANTIS scores for TCGA colorectal (CRC), endometrial (UCEC), and gastric (STAD) tumors showing cases with discordant classifications between the two conventional MSI callers. Each point represents a tumor sample with conflicting MSI status by the established thresholds (MSIsensor > 10 and MANTIS > 0.4; red dashed lines). Points are colored by their PROMIS score using a blue-yellow-red gradient, corresponding to low, intermediate, and high fractions of unstable loci.

Supplementary Figure 2. Scatterplots illustrating the relationship between tumor mutational burden and PROMIS score across TCGA colorectal (CRC), endometrial (UCEC), and gastric (STAD) cancers. **(A)** Correlation of PROMIS scores with mutation count and nonsynonymous TMB across all cases, including POLE-mutated samples. **(B)** Same analysis after exclusion of POLE-mutated tumors, showing the characteristic reduction of extreme outliers with ultra-high mutational load. Red dashed lines indicate the PROMIS classification threshold of 5.

Supplementary Figure 3. Repeat-unit distributions of microsatellite length changes across TCGA cancer types. Panels A-C show the mean within-tumor distributions of signed repeat-length shifts in colorectal (CRC), endometrial (UCEC), and gastric (STAD) cancers, respectively. Shifts were measured from the nearest stable-allele boundary and expressed in repeat units, with negative and positive values indicating contractions and expansions. Only changes corresponding to an integer number of repeat units were included. Panel D summarizes the mean within-tumor proportion of contractions. Lines and points represent equally weighted tumor-level means, while shaded ribbons and error bars indicate tumor-level bootstrap 95% confidence intervals. MSS tumors are shown as dashed lines with open circles and MSI tumors as solid lines with filled triangles.

Supplementary Figure 4: PROMIS score versus matched tumor-normal MSIsensor score in the multiregional metastatic phs001925.v1.p1 cohort. Each point represents one sample. Tumor samples are colored by patient and cancer type, and matched normals are shown in gray at an MSIsensor value of 0 to visualize their distribution along the PROMIS axis. The red dashed lines indicate the predefined classification thresholds for PROMIS (>5) and MSIsensor (>10). The black dashed line shows the linear regression fit across tumor samples only.

Supplementary Figure 5: Performance of PROMIS across genomic subsets and tumor fractions with the common relaxed configuration. A total of 513 tumors (233 MSI and 280 MSS) were analyzed at in silico tumor fractions of 10%, 5%, 3%, and 1%. using the relaxed common configuration to accommodate low tumor fractions. **(A)** AUC, reflecting threshold-independent separation of MSI and MSS scores. **(B)** Sensitivity and **(C)** specificity at PROMIS cutoffs of >10 for CRC and >5 for UCEC and STAD. **(D)** Number of instability-supporting reads. Each point represents one sample at the indicated tumor fraction; MSS and MSI samples are shown in the left and right subcolumns, respectively. Boxes indicate the median and interquartile range, and read counts are displayed on a logarithmic scale. Colors denote cancer type, whereas black lines in panels A-C denote pooled estimates. Error bars show 95% confidence intervals for the pooled estimates.

Supplementary Figure 6: Detailed PROMIS metrics across decreasing tumor fractions. A total of 513 tumors (233 MSI and 280 MSS) were analyzed at in silico tumor fractions of 10%, 5%, 3%, and 1%, using the relaxed common configuration for STAD and UCEC, and the stricter threshold for CRC. Pooled boxes are unfilled, whereas cancer-specific boxes are color-coded by tumor type. Boxes indicate the median and interquartile range, and whiskers extend to 1.5 times the interquartile range**. (A)** PROMIS score. **(B)** Number of evaluable microsatellite loci. **(C)** Number of loci classified as unstable. **(D)** Median number of instability-supporting reads per unstable locus. **(E)** Median percentage of reads supporting instability per unstable locus. **(F)** Composition of qualifying deviating reads according to their signed repeat-length shift relative to the dominant stable allele. Negative shifts indicate microsatellite shortening and positive shifts indicate lengthening. Bars show the percentage of reads in each shift category.

Supplementary Figure 7. Effect of recurrent unstable region filtering on PROMIS scores in TSO500 cfDNA samples. Paired comparison of unfiltered PROMIS scores and scores recalculated after excluding microsatellite loci classified as unstable in at least 30% of samples. Each line represents one sample and is colored by MSI status. Filtering recurrent unstable regions reduced MSS/background signal while largely preserving MSI-associated signal, supporting the interpretation that a subset of loci contributes panel-specific background instability in this high-coverage targeted assay.

Supplementary Figure 8. PROMIS scores in the exploratory Ion Torrent cohort. PROMIS scores are shown for nine samples with verified MSI annotations (7 MSS and 2 MSI), with each point colored according to MSI status. The dashed horizontal line marks the predefined PROMIS classification threshold of 5. Because several samples had very high PROMIS scores, the y-axis is displayed with a break to improve visualization of the lower score range. The substantial overlap between MSS and MSI samples indicates that PROMIS did not achieve robust discrimination in this Ion Torrent dataset and is not validated for this platform in its current form.
